# Global Atmospheric River Lifecycle Detection Using Integrated Water Vapor and Vapor Transport

**DOI:** 10.1038/s41597-025-06198-9

**Published:** 2025-12-08

**Authors:** Brandon Kerns, Shuyi S. Chen

**Affiliations:** University of Washington, Department of Atmospheric and Climate Science, Seattle, USA

**Keywords:** Atmospheric science, Natural hazards

## Abstract

Atmospheric rivers (ARs) are narrow streams of enhanced water vapor transport. While there is no accepted quantitative definition of ARs, integrated water vapor transport (IVT) is most commonly used to detect them. Nevertheless, narrow features of enhanced total precipitable water (TPW) are common signatures of ARs. Current AR tracking methods are generally limited to using either IVT or TPW separately, and many do not track the ARs globally through their life cycles. In this study, a global Atmospheric River Lifecycle Detection (ARLiD) method and 44-year database that incorporates both IVT and TPW are presented. The inclusion of TPW provides consistency in identifying entire lifecycle of ARs and reduces uncertainties due to small changes in wind. ADLiD extends the AR systems both equatorward and poleward of the IVT. The data are useful for studies that wish to include portions of the AR life cycle during which the AR, or portions of the AR, have a stronger signature in TPW than in IVT.

## Background & Summary

Atmospheric water vapor transport is a crucial link in Earth’s hydrological cycle. The origin of atmospheric water vapor is ultimately local air-sea fluxes from the ocean, with localized contributions from inland lakes and evapotranspiration. Due to the constant exposure of the sea surface to winds and the high carrying capacity of the tropical atmosphere, the tropics serve as a global moisture reservoir. Elevated tropospheric moisture and heavy rainfall outside of the tropics is commonly related to narrow moist filamentary structures referred to as atmospheric rivers (ARs) as opposed to broad fields of moisture transport^1–4^. It is estimated that 30–50% of total water vapor transport and 90% of meridional transport in the mid-latitudes is associated with ARs, despite them occupying on average ~10% of the longitudinal width^2^. In addition to meridional transport, moisture transport from the sea to land is important. ARs that impinge on mountainous coastal areas often produce copious rainfall^5–7^. The influence of ARs also extends inland, where they are important for water resources and can lead to impactful flooding in arid regions^8–10^.

There is currently no single accepted quantitative definition of ARs, and numerous methods have been devised to identify and track them. The first consideration is what variable(s) to use. ARs were originally identified in gridded model analysis using integrated water vapor transport (IVT)^1,2^. Subsequent methods using satellite data and model analysis were often based on total precipitable water (TPW)^5,11–16^. Nevertheless, most recent AR tracking methods use integrated water vapor transport IVT^17–22^. One exception is that for Antarctic latitudes, TPW has been used^23–25^. Some studies have considered both TPW and IVT based methods^17,25^; nevertheless, they did not incorporate both TPW and IVT into a single method.

**Fig. 1:**
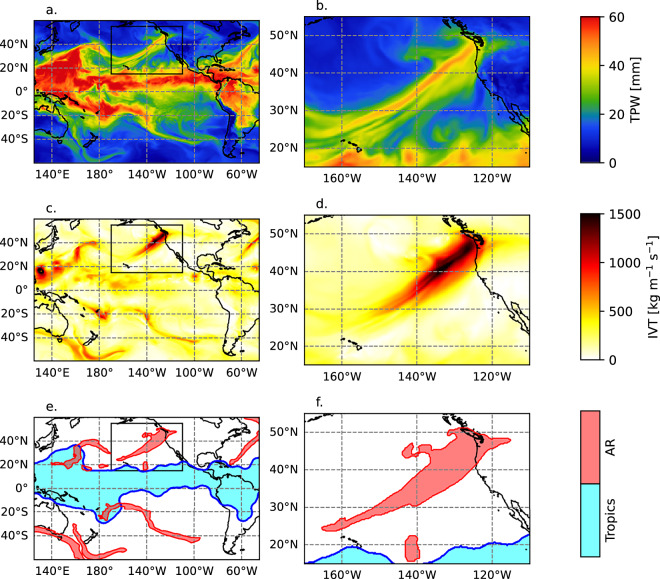
Illustration of the input variables and ARLiD classifications for 1200 UTC 20 Oct. 2003. Total precipitable water for the greater Pacific region (**a**) and the zoomed in northeast Pacific region (**b**). Integrated water vapor transport for the greater Pacific region (**c**) and the zoomed in northeast Pacific region (**d**). Identified atmospheric river systems (red) and deep tropics region (cyan) for the greater Pacific region (**e**) and the zoomed in northeast Pacific region (**f**). Panels (**b**), (**d**), and (**f**) are zoomed in to the boxes drawn in (**a**), (**c**), and (**e**), respectively.

Another limitation of many AR detections methods is that they are either implemented for specific regions or that they do not track the ARs in time through their life cycles. At the time of this article, there are 40 AR detection techniques (ARDTs) listed online (https://www.cgd.ucar.edu/projects/artmip/algorithms) by the Atmospheric River Tracking Method Intercomparison Project (ARTMIP)^26^. 14 of the methods are applied globally, and 11 of them track the ARs in time. However, only 4 methods are both global and tracked in time, and all of these 4 methods are based on IVT. These four methods are summarized in Table 1. The goal of this dataset is to track ARs in time, throughout their life cycles, globally, and to incorporate both TPW and IVT (see Fig. 1).

**Table 1:** Summary of the ARDTs in ARTMIP that provide global AR track data through the AR life cycles (first four rows) and Atmospheric River Lifecycle Detection (ARLiD, this study; last row).

Algorithm Name	Variables	Description
A15: TempestLR^22,33^	IVT	Blob identification and tracking. A Laplacian filter (9 point with a stencil radius of ~800 km) is applied to IVT to identify IVT ridges. Tracking in time based on area overlap without splitting or merging.
A11, A12, A13: CONNECT^43,44^	IVT	Objects are identified using fixed threshold values of IVT (300, 500, and 700 kg m^−1^ s^−1^)
A30: AR-CONNECT^40^	IVT	Objects are identified based on region growing segmentation starting from a core (IVT > 700 kg m^−1^ s^−1^) outward to a boundary (IVT = 300 kg m^−1^ s^−1^). Voxel connectivity in space (lon, lat) and time without splitting or merging.
A38: SCAFET^45^	IVT, precip	1000 km smoothing and a Hessian matrix shape index (SI) for IVT. Potential ARs are ridges and caps with SI > 0.375. IVT direction within 45 deg. of the ridge orientation. Precip > 1 mm day^−1^.
ARLiD^37^ (this study)	IVT, TPW	AR and TPW blobs are first identified separately based on contrast from the background then combined into AR objects. ITV blobs: Laplace of Gaussian filtered IVT < −7. TPW blobs: 10 mm above the local area. AR objects are tracked in time with splitting and merging.

The algorithm identifier is provided for ARTMIP ARDTs.

## Data and Methods

### Data

The AR identification and tracking uses the ECMWF Reanalysis 5 (ERA-5)^27,28^. ERA-5 is widely regarded as a reliable and comprehensive global atmospheric reanalysis product. Data are provided hourly on a regular 0.25° latitude/longitude grid. The data were obtained using the National Center for Atmospheric Research (NCAR) Research Data Archive (RDA) via the URL https://rda.ucar.edu/datasets/d633000/. Example data download scripts are included in the Github repository. These data are openly available. The following 2-D integrated fields are used: tcwv (total column water vapor, also referred to as total precipitable water, TPW); viwve (eastward component of integrated water vapor transport), and viwvn (northward component of integrated water vapor transport). These fields are vertically integrated from the surface to the top of the atmosphere (1 hPa). Using the ERA-5 variables viwve and viwvn, the magnitude of integrated vapor transport (IVT) is calculated as IVT = sqrt(viwve^2^ + viwvn^2^). Figure 1a,b shows an example of TPW and IVT for an AR event that caused record rainfall in the Seattle metro area.

### Tropical moisture extent

The largest tropospheric moisture structure on earth is the moist tropics. The tropics have a distinct bimodal distribution of moisture, with the moist portion encompassing a near-contiguous region of the equatorial zone, occasionally extending to higher latitudes, and a sharp latitudinal gradient between the moist tropics and subtropics (e.g., Fig. 1a). ARs are regarded as weather systems that transport moisture from the deep tropics to higher latitudes (tropical moisture export) and also gather subtropical water vapor through evaporation^2,29–32^. Therefore, it is necessary to distinguish ARs from moisture structures that are entirely contained within the moist tropics, such as monsoons and tropical cyclones.

**Fig. 2:**
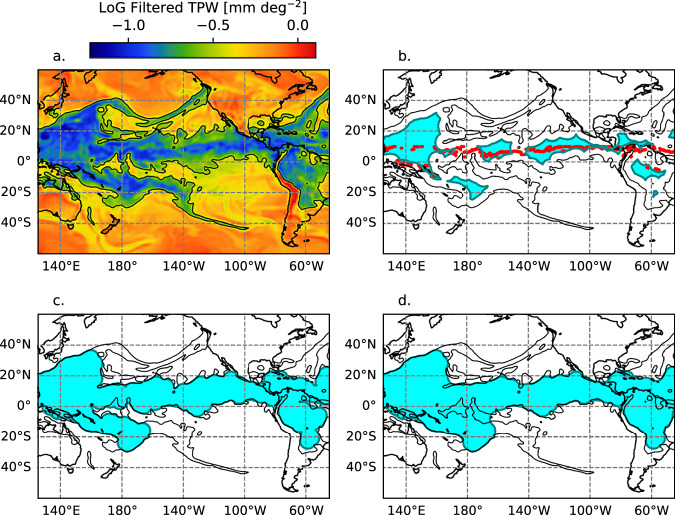
Illustration of the steps used to determine the deep tropics mask for 1200 UTC 20 Oct. 2003. (**a**) TPW with the Laplace of Gaussian filter applied (color shaded). For (**b**), (**c**), and (**d**), the cyan shading indicates the deep tropics mask identified at that step, as follows: (**b**) the first binary erosion step. The location of maximum TPW within 10°S–10°N for each longitude is indicated with the red dots; (**c**) after applying the binary dilation to the mask and the maximum TPW points in (**b**); and (**d**) the final mask is determined by applying an additional dilation then an erosion to the mask area in (**c**). In each panel, the unfiltered Laplace of Gaussian filtered TPW contour of −0.5 mm deg^−1^ is drawn in black.

The moist tropics mask is determined with the following algorithm. The boundary between of the moist air is determined using a Laplacian of Gaussian (LoG) filter and a dilation/erosion method. LoG filters are commonly used for edge detection in image processing. The standard deviation of the filter is 5.0°, and the filter is extended out two standard deviations. A threshold value of −0.5 mm deg^−2^ indicates the boundary of the moist air, including the deep tropics and ARs (Fig. 2a). However, the moist region includes the deep tropics and the ARs. The ARs appear as narrow extensions from the deep tropics. To separate out the deep tropics, an erosion – dilation method is used on the −0.5 mm deg^−2^ contour. This method is common in image processing to separate objects from sharp appendages that extend out from the object. The shape used for erosion and dilation is a circle with 5 deg. radius. First, a contraction is applied. This eliminates the ARs appendages, which generally have a diameter of <5 deg (Fig. 2b). However, it also eliminates portions of the deep tropics that are only a narrow ITCZ. To get the ITCZ back, the points corresponding to the maximum TPW within 10 S – 10 N along each longitude are added back (red markers in Fig. 2b). Then, a dilation is applied using the 5° circle shape, which expands the feature to encompass the deep tropics without the ARs (Fig. 2c). This feature often has artificially sharp concavities, which are removed by applying another dilation, followed by a contraction. Any holes left in the mask are filled in. Finally, any leftover portions that are separated from the deep tropics are removed by removing any portions of the deep tropics mask with area <1 × 107 km^2^. Figure 2d shows the deep tropics mask thus determined.

### Atmospheric river detection

**Fig. 3:**
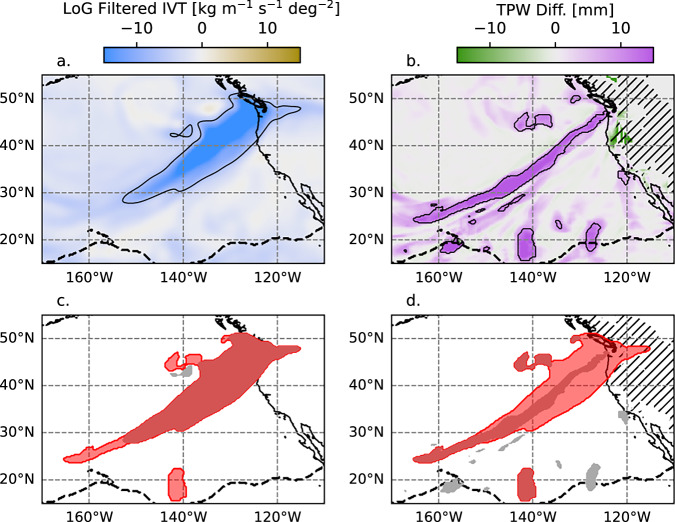
Construction of AR objects from the IVT and TPW blobs. (**a**) LoG filtered IVT with the blob threshold (−7 kg m^−1^ s^−1^ deg^−2^) outlined in black. (**b**) TPW difference from the local area background with the TPW blob threshold (10 mm) outlined in black. (**c**) IVT blobs (grey shading) overlaid with the AR objects (red shading). (**d**) TPW blobs (grey shading) overlaid with the AR objects (red shading). The portion of the AR system that is from the IVT (**c**) and TPW (**d**) blobs appears as darker red shading. In (**b**) and (**d**), topography >1000 m (excluded from TPW blobs) is indicated with the hatching. In all panels, the deep tropics mask is indicated with a black dashed curve.

AR objects are identified using a blob detection technique incorporating both IVT and TPW. First, IVT and TPW blobs are identified separately using the algorithms described for IVT and TPW below. These algorithms were applied to hourly ERA-5 data^22,33^. Second, the IVT and TPW blobs are combined into AR objects. AR objects consist of one or more IVT and/or TPW blobs. Figure 3 Illustrates the detection of IVT blobs, TPW blobs, and ARs. The blob detection was implemented using the Scipy ndimage library with a modification that allows for blobs to span across the prime meridian.

### IVT blob detection

The IVT blobs were detected by applying the same LoG filter used for deep tropics detection to the IVT field. Unlike TPW, the filtered IVT does not have a persistent, dominant maximum in the tropics. The threshold of filtered IVT used for blob identification is −7 Kg m^−1^ s^−1^ deg^−2^. (Fig. 3a). In the Indo-Pacific region, there is often a large area of persistently high IVT in the tropics, directed mainly zonally. This is related to large scale circulation patterns within the deep tropics rather than ARs. To filter this feature out, areas within the deep tropics mask with viwve > 250 kg m^−1^ s^−1^ are excluded from IVT blobs. Additionally, strong trade wind surges can result in a broad area of strong IVT. Therefore, areas within the deep tropics mask with viwve < −250 km m^−1^ s^−1^ are also excluded. Zonal IVT of magnitude >250 kg m^−1^ s^−1^ is rare in the tropics, outside of monsoons, strong trade winds, and tropical cyclones. For each time step, spatially contiguous regions satisfying the IVT criteria were tagged as IVT blobs (e.g., dark red shading in Fig. 3c).

### TPW Blob detection

ARs are narrow, elongated structures of enhanced moisture (TPW) and/or moisture transport (IVT). At first, an attempt was made to use the LoG filter to detect TPW blobs the same way as IVT. While the LoG filter was useful for detecting the deep tropics mask, the strong meridional gradient in TPW makes it difficult to use the LoG for elongated ridges. Therefore, a separate method was devised to identify TPW blobs as potential ARs.

TPW blobs are defined as elongated TPW structures that stand out from the background TPW. The background TPW is determined independently for each grid point as follows. First, local zonal and meridional transects were extracted from the TPW data for each grid point. The transects were 10° longitude and 10° latitude wide, respectively, with the grid point of interest at the center of each transect. Four background TPW values were calculated for the 10° transect to the north (TPW_bg_N), south (TPW_bg_S), east (TPW_bg_E), and west (TPW_bg_W). For points within 10° latitude of the North (South) Pole, the north (south) transect extended to the North (South) Pole. These four directional background values are the minimum TPW along each directional transect, excluding points where the topography is >1000 m. The final background TPW (TPW_bg) was calculated as: TPW_bg = MIN(MAX(TPW_bg_N, TPW_bg_S), MAX(TPW_bg_E, TPW_bg_W)). Using MAX(TPW_bg_N, TPW_bg_S) for the latitudinal transects ensures that TPW blobs are not detected merely based on low TPW at polar latitudes. TPW blobs were identified where the TPW - TPW_bg > 10 mm or TPW - TPW_bg > 0.5 X TPW_bg. (Fig. 3b). Note that if the TPW stands out from the background in either the longitudinal or latitudinal direction, it is considered to meet the TPW blob criterion. To avoid points meeting the criterion due to topography, TPW_bg values where the topography is ≥1000 m (hatching in Fig. 3b and 3d) were set to the original TPW, so that the differences would be zero. Additionally, these points were excluded from consideration as TPW blobs.

### Combining the TPW and IVT blobs into AR objects

The locus of points within either IVT blobs or TPW blobs was used to construct AR objects (lighter shading in Fig. 3c,d). Most AR objects consist of both IVT and TPW blobs; however, at times, the IVT falls below the threshold, while a TPW blob is detected, or vice-versa. Similar to IVT and AR blobs, the AR object detection is implemented using Scipy ndimage library with a modification that allows for blobs to span across the prime meridian. A minimum area criterion of 120,000 km^2^  is applied. Potential AR objects that were smaller than the area threshold were discarded.

Many IVT and TPW blobs ware detected entirely within the deep tropics region that are not considered to be ARs (e.g., the ITCZ and tropical cyclones). To filter them out, only AR objects that had an area extending outside of the deep tropics mask of at least 60,000 km^2^ (half of the total area criterion) were retained. No other morphological criteria (e.g., length/width and aspect ratio) were applied.

For each AR object, centroid latitudes and longitudes were calculated as area-weighted means. Typically, longitude values ranged from 0 to 360°, with 180 to 360° representing the Western Hemisphere. This range of longitude values also applied to the centroid longitudes. However, for AR objects spanning the prime meridian (0°E), longitudes were expressed on the −180 to 180° scale, where −180 to 0° corresponds to the Western Hemisphere. When most of the AR object was in the Western Hemisphere, the centroid longitude was negative. This approach prevents large jumps in centroid longitude values when tracking AR systems crossing the prime meridian (see below).

### Tracking atmospheric rivers in time

Similar to other AR tracking methods, AR objects were first identified using the detection methods laid out above. Then, the AR objects were connected in time as AR systems. The tracking method is adopted from Large-Scale Precipitation Tracking (LPT)^34,35^, but modified to allow for system splitting and merging. In order to be connected in time, AR objects at consecutive times (hourly) were required to overlap by a minimum of at least 20% of the area of either AR object, or 120,000 km^2^ , which is the minimum AR object area. Only AR systems that can be tracked for at least 48 h were included in the database.

**Fig. 4:**
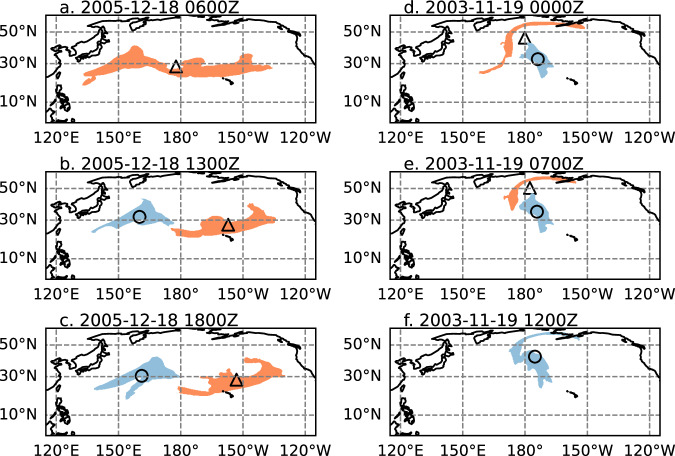
Example of splitting (**a**–**c**) and merging (**d**–**f**) AR systems. The orange and blue shading indicate the areal extent of the AR systems at the times indicated in the panel labels. The centroid locations of the AR systems are indicated by the circle and triangle marker.

AR systems often occur as splitting and merging systems in spatially contiguous groups, referred to as AR families. Splitting and merging were handled as follows. Splitting occurs when two or more AR objects at time t + 1 meet the overlapping criteria with the object present at time t. In this case, the largest intersecting object at time t + 1 was used to continue the AR system, and the others were used to initiate a new AR system track (e.g., Fig. 4a–c). Similarly, merging occurs when two or more AR objects present at time t meet the overlapping criteria with a single AR object present at time t + 1. For merger cases, the object at t + 1 continued the track of the AR system which was largest at time t, and the other overlapping AR systems present at t were terminated (Fig. 4d–f). AR systems arising from splitting or merging must each meet the 48-h duration criterion.

**Fig. 5:**
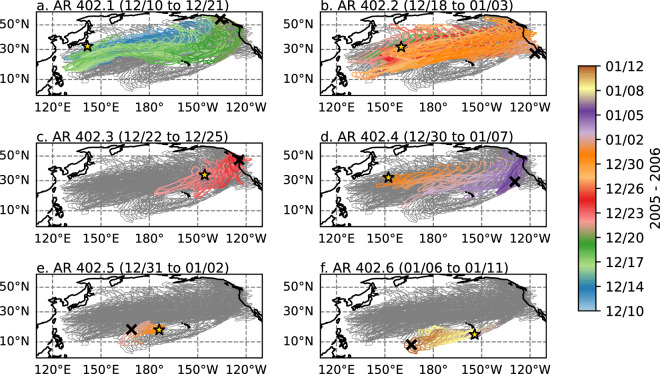
Time evolution of six individual AR systems as part of an AR family (#402 for the 2005–2010 tracking period) occurring in the North Pacific Ocean during 10 December 2005–12 January 2006. Each contour represents the boundary of the tracked AR system at a single time. Contours are drawn for every 6 h. The contour colors represent the time according to the colorbar. In the background, the contours for all AR systems in the family are drawn in grey. Initiation centroid points are indicated with star markers. Dissipation centroid points are indicated with X markers.

AR Families consist of one AR system (in case of no splits or mergers) or multiple AR systems that are related to each other due to merging and/or splitting (Fig. 5). Note that in methods using TempestExtremes, AR families are detected only as a whole and not as separate merging and splitting systems^22,36^. In the database, the AR families are denoted by an AR family number for each tracking year, and AR systems are indicated by decimal values with the same whole number parts. Figure 5 illustrates 6 individual AR systems associated with a long-lived (32 days) AR family (number 402 for the 2005–2006 tracking year) which occurred over the North Pacific Ocean during December 2005–January 2006. The AR family was tracked as an entity from 10 December 2005 to 11 January 2006. Within this AR family, three of the AR systems were tracked propagating eastward across the North Pacific for over a week: 402.1 (Fig. 5a, 11 days), 402.2 (Fig. 5b, 16 days), and 402.4 (Fig. 5d, 8 days). AR system 402.3 (Fig. 5c) formed closer to the US west coast, and it was tracked moving eastward for 3 days. Finally, AR systems 402.5 and 402.6 (Fig. 5e,f) were short lived systems which tracked westward at lower latitudes.

## Data Records

The data are provided in a public Zenodo repository^37^: “ARLiD: Atmospheric River Lifecycle Detection”^37^. The data are released under the Creative Commons Attribution 4.0 International license. The current version is Version 1. The repository can be accessed online at https://zenodo.org/records/16787789. The repository contains three sets of files for each tracking year. The tracking years run from 1 June to 30 June the following year, and the time period of tracking is indicated in each file name. To account for AR systems potentially being broken up at the beginning or end of the tracking year, there is overlap (1–30 June) between consecutive years. For example, tracking year 2020 covers 0000 UTC 1 June 2000 to 2300 UTC 30 June 2001, and this is indicated in the file names as “2020060100_2021063023.”

The data are provided in text (ASCII) and Network Common Data Format (NetCDF) formats. For each tracking year, there is a set of four data files: IVT and TPW blob files (NetCDF), two files (text and NetCDF) for the AR system centroid tracks and properties of the systems as a whole, a file indicating the grid points associated with each individual AR system (NetCDF), and a spatio-temporal mask file including the grid points of all AR systems at all times (NetCDF). The content of the four data files is summarized in Table 2, and more information is provided in the NetCDF metadata and the full variable descriptions in Zenodo.

**Table 2:** Summary of the data provided in each file.

File Name	Description of Data
ar_blob_data_YYYY.tar.gz	Compressed tar archives of NetCDF files containing the deep tropics mask, TPW blobs, IVT blobs, and potential AR objects at each time.
lpt_systems_ar_{TIMES}.txt	Human readable AR systems information. For each AR system, a table is provided with the area, centroid latitude, centroid longitude, and number of AR objects at each time.
lpt_systems_ar_{TIMES}.nc	Detailed AR systems information in a structured NetCDF format. In addition to the variables in the.txt file, the bounding box of the AR systems at each time is given. A list of grid points corresponding to each individual AR system is provided for each time through its life cycle.
lpt_composite_mask_{TIMES}.nc	Spatio-temporal mask of all AR systems combined. Binary data: 0 = no AR, 1 = AR.

{TIMES} indicates the time period for each file in the format YYYYMMDDHH_YYYYMMDDHH, for example, 2020060100_2021063023 for the 2020 tracking year. The ar_blob_data files are for the calendar years.

## Technical Validation

The ARLiD data^37^ have been validated by comparing the track initiation, dissipation, and AR frequency of occurrence with several ARDTs used in the ARTMIP. ARTMIP Tier 2 catalogue data were used for this comparison^38^. Comparisons were made with three ARDTs: SCAFET, TempestLR, and AR-CONNECT. The underlying data for SCAFET was the Modern-Era Retrospective analysis for Research and Applications, Version 2 (MERRA2). For TempestLR and AR-CONNECT, the underlying data was ERA-5. Because previous studies have mainly tracked the AR families as a whole, without splitting and merging, the results for AR families are used to make comparisons with the other ARDTs.

**Fig. 6:**
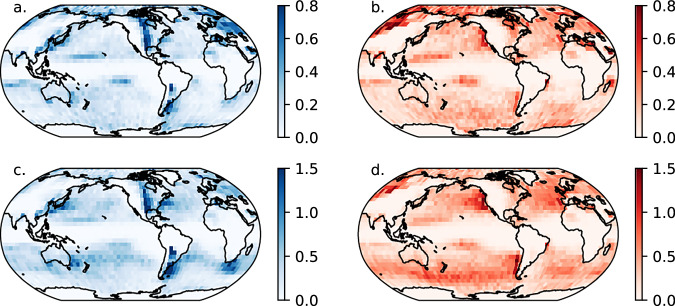
Initiation and dissipation density of ARs for June 1980 – June 2024. (**a**) Initiation of AR families; (**b**) Dissipation of AR families; (**c**) Initiation of individual ARs, and (**d**) dissipation of individual ARs. The units are count of AR centroids per year per 100,000 km^2^. Bins are 5 × 5 deg.

Initiation and dissipation of ARs tends to be concentrated in distinct favorable regions (Fig. 6). The initiation and dissipation of AR systems (Fig. 6a,b) is generally more spread out than for AR families (Fig. 6c,d), since AR systems often form due to splitting or dissipate due to merging during the life cycle of an AR family. Previous studies show that ARs tend to form in the western portions of ocean basins and dissipate in the eastern portions, usually at latitudes poleward of the initiation locations^39–41^. In ARLiD, this lifecycle behavior is similar. AR families and AR system initiation is favored off the east coasts of Asia, North America, Australia, and South America (Fig. 6a,c). Another favorable initiation point for AR families is to the south of southern Africa. Additionally, initiation is locally favored east of the Rocky Mountains in North America and the South American Andes. This localization is reflected in previous studies^40^. Meanwhile, dissipation is favored in the eastern portions of the North and South Pacific Ocean, off the coasts of North and South America (Fig. 6b,d). Over the North Atlantic and the Southern Ocean, dissipation of ARs is more spread out. One feature of ARLiD that is not reflected in many previous studies is at there are favored bands of both initiation and dissipation at low latitudes in the western North and South Pacific. These are relatively short-lived systems that dissipate near their initiation locations, which have been found with regional AR tracking for the North Pacific^20^. It is beyond the scope of this study to determine their relationships with more “classic” AR systems further poleward, although some of them may be AR systems that break off from AR families at lower latitudes and propagate westward (e.g., Fig. 5e,f).

**Fig. 7:**
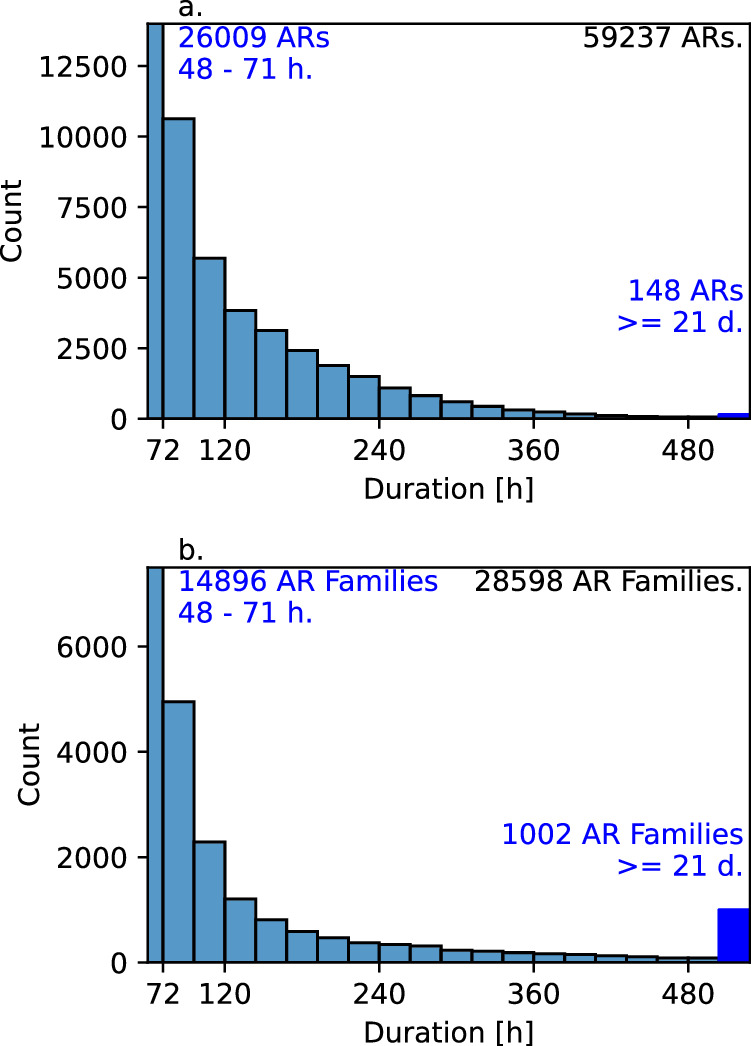
Histogram of AR duration for June 1980 – May 2024. (**a**) Duration of individual AR systems. (**b**) Duration of AR families. Bins are every 24 h, inclusive on the left and exclusive on the right. The counts of total systems is given in the upper right. The count of systems with duration 48–71 h is at the upper right. The count of systems with duration >=21 days is at the lower right, and the rightmost bar includes all systems with duration >=21 days.

AR systems and families occur across a range of time scales from days to weeks, with durations < 1 week (e.g., synoptic scale) most common (Fig. 7). There are some particularly long lived ARs in the database. 7% of AR families and 0.6% of AR systems had durations of >=3 weeks. The longest-lived AR families occur over the Southern Ocean (not shown). Other AR tracking methods also produce ARs with durations of several weeks, and even several months^40,42^. Assessing the duration of ARs globally remains a challenge. The duration of tracked ARs varies significantly depending on the detection criteria and tracking method.

**Fig. 8:**
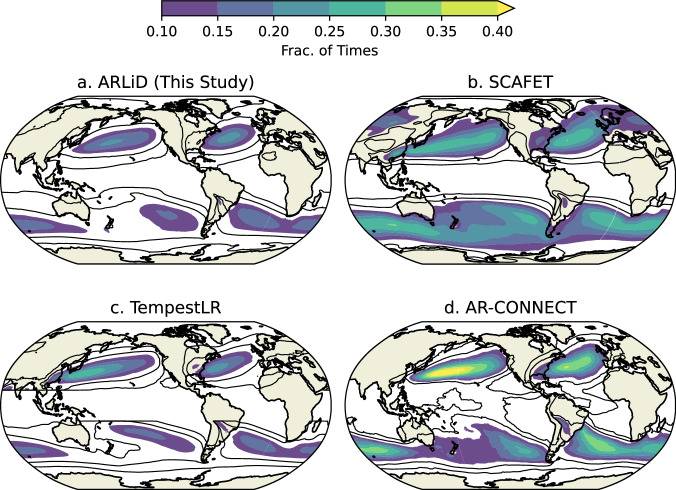
Comparison of AR frequency of occurrence for (**a**) ARLiD (this study), (**b**) SCAFET, (**c**) TempestLR, and (**d**) AR-CONNECT. See Table 1 for more details regarding the products.

Figure 8 shows a comparison of AR frequency of occurrence compared with three other global AR datasets. To calculate the frequency of occurrence, a spatio-temporal mask of AR systems was constructed. The frequency of occurrence is defined at each grid point as the number of times (hourly) for which an AR system encompassed the grid point, divided by the total number of times. For the ARTMIP products, the frequency of occurrence was calculated using the spatio-temporal mask data provided by the ARTMIP website. Frequency of occurrence is a function of the number of AR systems tracking in the vicinity, the size of the AR systems, and the propagation speed. In general, AR frequency is maximized at sub-tropical to polar latitudes (e.g., 30–60°N or °S) over the ocean. The frequency of occurrence of AR systems in this study is on the lower range compared to other ARTMIP products in Table 1. Since the method is most similar to TempestLR, is it encouraging that the frequency of occurrence in ARLiD is similar to TempestLR (Fig. 8a,c).

## Usage Notes

Most tracked AR systems are narrow, elongated features that propagate eastward in the mid-latitudes. Nevertheless, there are some relatively small, short-lived features at lower latitudes with westward propagation, especially in the central and western North Pacific. While these have generally not considered to be ARs in previous studies, they are retained in the database for completeness. Depending on the user’s objectives, it may be beneficial to exclude these systems from their analysis. Additionally, the data do not exclude tropical cyclones in any explicit way. Tropical cyclone best track data may be useful to filter out AR systems that are connected to tropical cyclones.

The tracking periods are in 5-year increments, starting on 1 June and ending in 31 May at the end of the 5-year period (e.g., 1 June 2005–31 May 2010). When analyzing multiple years, it may be important to consider that some AR systems are broken up at the end of the 5-year increment. This can be accomplished in two ways: 1.) Disregard all the AR systems that begin at 0000 UTC 1 June of the beginning year and those that end at 2300 UTC 31 May of the ending year; or 2.) Use spatial overlap to connect the AR systems between two consecutive 5-year tracking periods. For Figs. 6 and 7, we have taken the former approach.

## Data Availability

The data are provided in a public Zenodo repository^37^: “ARLiD: Atmospheric River Lifecycle Detection”^37^. The repository is at https://zenodo.org/records/16787789. The data are released under the Creative Commons Attribution 4.0 International license.

## Code availability

The Python code used to generate the AR objects files is provided in a public GitHub repository: https://github.com/uwincm/arlid. A tag “scidata_v1” is created for the version of code used to generate the data for the scidata_v1 data on Zenodo. The code used to implement the AR identification and tracking is the same used for Large Scale Precipitation Tracking (LPT) of the Madden-Julian Oscillation^34,35^. The LPT code (with tag “arlid_scidata_v1”) is linked to the ARLiD repository as a submodule. The LPT code is available on Github at https://github.com/uwincm/lpt-python-public/tree/arlid_scidata_v1. The configuration settings used to run the LPT and generate AR systems data from the AR objects files are provided in the ARLiD Repository.
